# Flexible torsion-angle noncrystallographic symmetry restraints for improved macromolecular structure refinement

**DOI:** 10.1107/S1399004714003277

**Published:** 2014-04-30

**Authors:** Jeffrey J. Headd, Nathaniel Echols, Pavel V. Afonine, Nigel W. Moriarty, Richard J. Gildea, Paul D. Adams

**Affiliations:** a Lawrence Berkeley National Laboratory, Berkeley, CA 94720, USA; b Duke University Medical Center, Durham, NC 27710, USA; c Diamond Light Source, Harwell Science and Innovation Campus, Didcot OX11 0DE, England; d Department of Bioengineering, UC Berkeley, Berkeley, CA 94720, USA

**Keywords:** macromolecular crystallography, noncrystallographic symmetry, NCS, refinement, automation

## Abstract

One of the great challenges in refining macromolecular crystal structures is a low data-to-parameter ratio. Historically, knowledge from chemistry has been used to help to improve this ratio. When a macromolecule crystallizes with more than one copy in the asymmetric unit, the noncrystallographic symmetry relationships can be exploited to provide additional restraints when refining the working model. However, although globally similar, NCS-related chains often have local differences. To allow for local differences between NCS-related molecules, flexible torsion-based NCS restraints have been introduced, coupled with intelligent rotamer handling for protein chains, and are available in *phenix.refine* for refinement of models at all resolutions.

## Abstract 2

Flexible torsion angle-based NCS restraints have been implemented in *phenix.refine*, allowing improved model refinement at all resolutions. Rotamer correction and rotamer consistency checks between NCS-related amino-acid side chains further improve the final model quality.

## 1. Introduction   

One of the great challenges in macromolecular crystallo­graphy is improving the data-to-parameter ratio. When crystals contain multiple copies of the same molecule or complex within the asymmetric unit, it is reasonable to assume that these related entities will generally adopt similar, if not identical, conformations. These noncrystallographic symmetry (NCS) relationships have been used previously to address the phasing problem (Rossmann, 1972 ▶; Bricogne, 1976 ▶). For refinement, restraints that maintain similarity between related atomic positions can be added to the geometry target function, introducing correlations between refined parameters. Alternatively, constraint-based approaches improve the data-to-parameter ratio by requiring NCS-related regions to be identical, such as the methods described in Hendrickson (1985 ▶) and Kleywegt (1996 ▶). However, the use of constraints often inappropriately enforces structural identity where there are local structural differences, which is particularly observable at high resolution (∼1.8 Å or better). To address this issue, NCS restraints for structure refinement have previously been implemented in a variety of crystallographic refinement programs, including *PROLSQ* (Hendrickson, 1985 ▶), *TNT* (Tronrud *et al.*, 1987 ▶), *FROG* (Urzhumtsev *et al.*, 1989 ▶), *CNS* (Brünger *et al.*, 1998 ▶), *SHELX* (Sheldrick, 2008 ▶), *BUSTER* (Bricogne *et al.*, 2010 ▶), *REFMAC* (Murshudov *et al.*, 2011 ▶) and *phenix.refine* (Afonine *et al.*, 2012 ▶).

Many past implementations of NCS restraints enforced global similarity between groups, effectively treating the symmetry relationships as rigid. Even at moderate resolution, however, differences between NCS-related copies are often supported by crystallographic data, and must be taken into account to maximize the effectiveness of such restraints without overfitting of the data by the model (Usón *et al.*, 1999 ▶). One may simply remove NCS restraints for groups with clear conformational difference, but this approach typically requires careful manual inspection of the model and maps, and is not easily automated. Moreover, the inflexibility of global restraints during refinement prevents convergence when starting from identical copies, for instance after molecular replacement. Alternative approaches have been implemented for NCS restraints, which instead restrain local conformation (Usón *et al.*, 1999 ▶; Sheldrick, 2008 ▶). More recently, local NCS implementations in both *REFMAC* (Murshudov *et al.*, 2011 ▶) and *BUSTER* (Smart *et al.*, 2008 ▶, 2012 ▶) use local similarity restraints based on distances between atoms nearby in space. In each implementation, these restraints resemble a simple harmonic near the target values, tapering off to no restraint as the distance increases, similar to the reference-model restraint implementations in* BUSTER* (Smart *et al.*, 2012 ▶), *REFMAC*5 (Nicholls *et al.*, 2012 ▶) and *phenix.refine* (Headd *et al.*, 2012 ▶).

Here, we discuss an implementation of local similarity-based NCS restraints that use torsion angles rather than local atomic distances. Torsion angles are chosen for their well understood relationship to macromolecular folding, *i.e.* correlated ϕ/ψ conformations (Ramachandran & Sasisekharan, 1968 ▶), amino-acid side-chain rotamers (Lovell *et al.*, 2000 ▶), and RNA backbone conformations (Richardson *et al.*, 2008 ▶) *etc.*, which allow a limited number of restraints to govern the coordinated movement of related structural elements.

## 2. Methods   

Torsion NCS restraints in *phenix.refine* are implemented using the same torsion-based ‘top-out’ potential as described in Headd *et al.* (2012 ▶). Briefly, a torsion restraint is added for each NCS-related torsion angle in the working model. In proteins, this set of angles includes all protein side-chain χ angles and the backbone ϕ, ψ and ω angles. Improper dihedral C—N—C^α^—C^β^ and N—C—C^α^—C^β^ restraints are also added for each protein residue to preserve C^β^ geometry, with each torsion restrained to the ideal value for the given residue type (Lovell *et al.*, 2003 ▶). For nucleic acids, this set of torsion angles includes all seven backbone torsions, as well any defined base χ angles. Only macromolecules (protein and/or nucleic acid) are handled in the current implementation. Non-standard amino acids and nucleic acids are also supported automatically. It should also be noted that explicit torsion restraints are an improvement over the 1,4-distance-based approach described in Usón *et al.* (1999 ▶), resolving the 180° ambiguity that exists for some 1,4 distances, such as χ^2^ for the **p90** and **p−90** Trp rotamers.

As discussed in Headd *et al.* (2012 ▶), the ‘top-out’ potential is defined by σ and limit parameters, with the latter parameterized in degrees to control at what difference between related torsions the target is smoothly reduced to zero. Our implementation is similar to the Welsch robust estimator function (Dennis & Welsch, 1978 ▶), and is conceptually similar to the local NCS potentials implemented in *REFMAC*5 (Murshudov *et al.*, 2011 ▶) and *BUSTER* (Smart *et al.*, 2012 ▶). The target for each set of NCS-related torsions is defined to be their average (except as noted below), which is updated after each refinement step that moves individual sites, including real-space refinement, reciprocal-space refinement and Asn/Gln/His side-chain orientation correction. The residuals for the torsion NCS restraints are calculated using the following ‘top-out’ functional form:
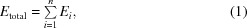


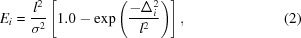
where Δ*_i_* is the difference between the *i*th torsion and its NCS-related average, σ is a user-definable standard deviation parameter, *l* is the limit parameter and *n* is the total number of added reference restraints. It should be noted that the average of two torsion angles is calculated by taking the tangent of the quotient of their average sines and cosines.

Atomic displacement parameters (ADPs) for NCS-related atoms may be restrained using the same parameterization as for global, Cartesian-based NCS restraints, but we have found that allowing the ADPs to be refined independently typically results in improved *R* factors (data not shown), as NCS-related chains will often have considerably different ADPs. This observation is consistent with previous reports (Smart *et al.*, 2012 ▶), and is also supported by the variation in TLS (translation, libration, screw-rotation) models observed for NCS-related chains in some cases (Burnley *et al.*, 2012 ▶).

For this manuscript, all refinements in *phenix.refine* were carried out using *Phenix* v.1.8.4-1496.

### 2.1. Knowledge-based rotamer correction for protein side chains   

Torsion-angle parameterization of NCS restraints allows the inclusion of additional prior knowledge of protein geometry. To this end, we identify the rotameric state of each protein side chain (standard amino acids only), and only restrain matching χ angles that are in the same rotameric state. For side chains with multiple χ angles (such as Lys), we match successive χ angles out from the main chain as long as they match, while not restraining any χ angles past the first angle that differs. For example, if two matching Lys residues were in **mmtt** and **mmmt** rotamer states (for a discussion of rotamer nomenclature, see Lovell *et al.*, 2000 ▶), respectively, only χ_1_ and χ_2_ would be restrained. Parameterizing the restraints in this manner avoids restraining the χ_4_ angle, which may have a similar torsion angle but is in a different rotamer state. Further, we do not allow rotamer outliers, Ramachandran outliers (ϕ and ψ angles) or peptide outliers (ω angle >30° or <−30°) to contribute to NCS target values. If such outliers are matched to one or more NCS-related torsions with an allowed conformation, then the target value is calculated from the set of allowed values, and the outlier is restrained to this value. If during the course of refinement these outliers are resolved to allowed conformations, their torsion values will contribute to the NCS average target calculation during the next macro-cycle (Afonine *et al.*, 2012 ▶).

#### 2.1.1. Rotamer correction   

**Figure 1:**
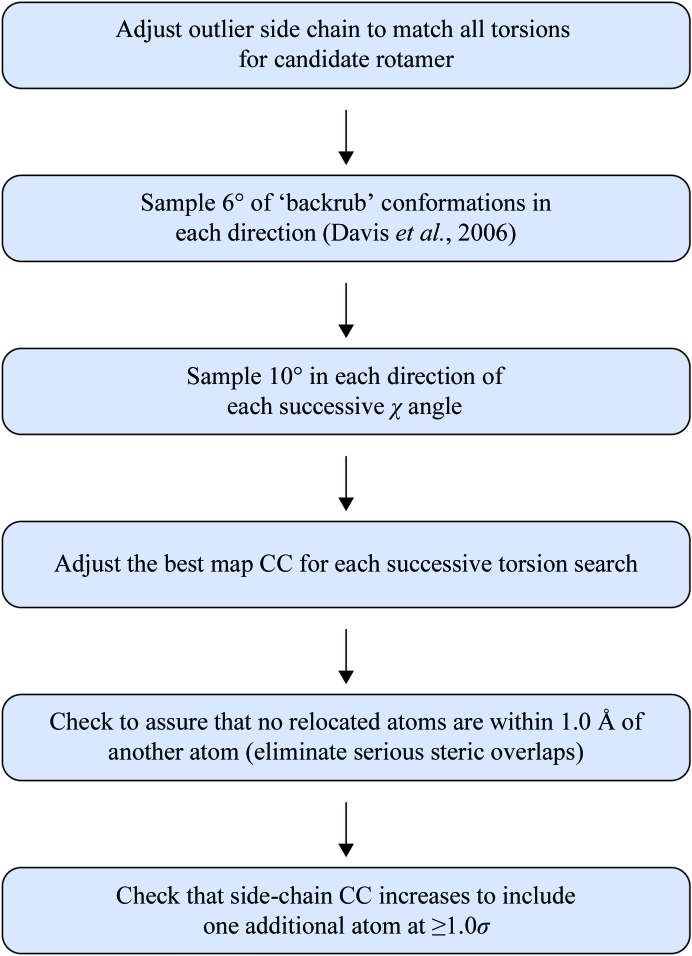
Flow diagram of NCS-related automated rotamer correction.

For proteins, knowledge of side-chain rotamer distributions (Lovell *et al.*, 2000 ▶) can be used to identify and correct rotamer outliers at each macro-cycle. At high resolution (roughly 1.8 Å or better), density shape alone is often sufficient to correctly fix rotamer errors. As shown in Headd *et al.* (2009 ▶), however, the ability to confidently accept such corrections drops off sharply below 2.5 Å resolution. Using the knowledge of NCS, we can limit the scope of rotamer searching to only attempt to fit rotamer states observed in NCS-related copies of the same residue. By limiting the search space, we are able to extend our ability to accept or reject candidate corrections at lower resolution (as low as 3.0 Å by default) through *a priori* knowledge of what the conformation should be. While the scope of this search would miss the case where all side chains in an NCS-related set should be an unrepresented rotamer, it also limits errors that could arise by introducing new rotamer possibilities that are not supported by NCS information. The side-chain fitting protocol is similar in concept to the ‘Autofix’ routine presented in Headd *et al.* (2009 ▶), but is limited to a 6° ‘backrub’ search (Davis *et al.*, 2006 ▶) followed by progressive 10° χ-angle sampling. The backbone conformation must be sampled to correct any backbone distortions brought about by the incorrect starting orientation. The ‘backrub’ is described as a rotation which rotates all atoms between two flanking C^α^ atoms as a rigid body. Such motions have been shown to be necessary in conformational sampling in protein design (Georgiev *et al.*, 2008 ▶; Keedy *et al.*, 2012 ▶). The full protocol is shown in Fig. 1 ▶. Corrections are rejected if any significant steric clashes are introduced with the surrounding model, and it is also required that at least one additional side-chain atom has a real-space correlation coefficient greater than or equal to 1.0σ. These strict requirements provide greater confidence that corrected rotamers are a reasonable steric fit for the model, as well as an improved fit to the experimental data.

#### 2.1.2. Rotamer consistency   

In cases where all NCS-related side chains are rotameric, but not all in the same rotamer state, it is possible that one or more side chains are misfitted. To identify and correct such cases, each side chain is tested against all possible candidate rotamers, with the best fit being accepted in each case following the above protocol.

**Figure 2:**
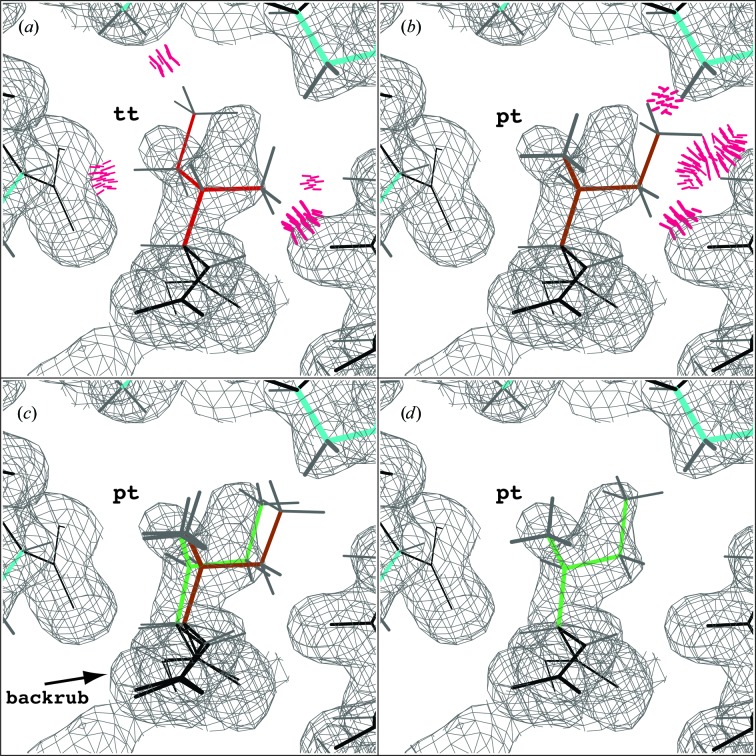
NCS rotamer consistency correction for Ile*A*120 in PDB entry 1m0o at 1.7 Å resolution. (*a*) Starting orientation of Ile*A*120 in 1m0o in the **tt** rotamer. Bad steric clashes (>0.4 Å) are depicted in hot pink. (*b*) χ angles adjusted to match the **pt** rotamer orientation of the NCS-related Ile*B*120 side chain. (*c*) Result of the local conformation search, including backrub motion, shown in green. (*d*) Following a default run of *phenix.refine*, the correct **pt** rotamer is fitted to the density map. All maps are 2*mF*_o_ − *DF*_c_ maps contoured at 1.2σ. Images were generated using *KiNG* (Chen *et al.*, 2009 ▶).

To test the effectiveness of rotamer outlier and consistency correction, we re-refined the 1.7 Å resolution triosephosphate isomerase structure (PDB entry 1m0o; Symersky *et al.*, 2003 ▶), which consists of two NCS-related copies in the asymmetric unit. In the deposited model, the Ile*A*120 side chain has been incorrectly fitted as a **tt** rotamer. As shown in Fig. 2 ▶(*a*), the **tt** conformation is not a good fit to the 2*mF*_o_ − *DF*_c_ map (see Afonine *et al.*, 2012 ▶ for map details) and has significant steric clashes with neighboring residues. The NCS-related Ile*B*120 residue, however, is fitted as a **pt** rotamer and is an excellent fit to both the density map and the local steric environment (data not shown). The automated rotamer consistency method first rotates the χ angles of Ile*A*120 to match those of the **pt** rotamer observed for Ile*B*120 (Fig. 2 ▶ *b*) and performs a local χ-angle and backrub conformation search (see above and Fig. 2 ▶ *c*) in order to determine whether this is a likely conformation. After reciprocal-space refinement, the correct **pt** rotamer proves to be an improved fit to the density map and the local environment (Fig. 2 ▶ *d*). Similarly, the Asp*A*32 side chain, which begins in an outlier conformation, is corrected to the **m−20** rotamer conformation of Asp*B*32 using the same local search method (data not shown).

### 2.2. Human-readable output   

When refining against a target that includes an adaptive geometric restraint, such as our flexible torsion NCS restraints, we note that it is important to provide informative feedback to the user about how such restraints were applied in a given refinement in order to quantify its impact on the resultant model. To this end, we provide summaries of all applied torsion NCS restraints in the comprehensive geometry restraints (GEO) files (available both before and after refinement). We also provide a residue-by-residue matching summary for matched residues, as well as step-by-step updates on the number of active torsion NCS restraints. Finally, we provide an NCS summary to the output PDB header, which includes details of each NCS group, the number of matched torsions in a given group and torsion-based r.m.s.d. values for all torsions above and below the set limit value. We also include a histogram of torsion-angle differences between the model and target for those torsions above and below the limit.

Providing such information allows detailed explanation of methods in structure publications where torsion NCS restraints are used. Through the *iotbx.cif* routines (Gildea *et al.*, 2011 ▶), *phenix.refine* is also fully compliant with migration to mmCIF format (Westbrook & Fitzgerald, 2005 ▶), and will allow the propagation of explicit NCS restraint information.

### 2.3. Refinement at medium–high resolution: an in-depth example   

Because the torsion-based NCS restraints allow for local differences, including rotamer differences between NCS-related side chains, these restraints can be safely used even at high resolution without much risk of negatively impacting the refinement. To test the efficacy of our restraints in a real experimental context, we performed molecular replacement (MR) in *Phaser* (McCoy *et al.*, 2007 ▶) with the data for RNAse S (PDB entry 1sar; Sevcik *et al.*, 1991 ▶), which are nominally at 1.8 Å resolution (>90% complete to 1.85 Å resolution) using the *A* chain from a related 2.0 Å resolution RNAse S structure (PDB entry 1rsn; Sevcik *et al.*, 2005 ▶) as a search model. Crystallographic symmetry operators and origin shifts were applied to the resultant MR solution using *phenix.find_alt_orig_sym_mate* (Oeffner *et al.*, 2012 ▶) to place the model in the same frame of reference as the deposited 1sar model.

**Figure 3:**
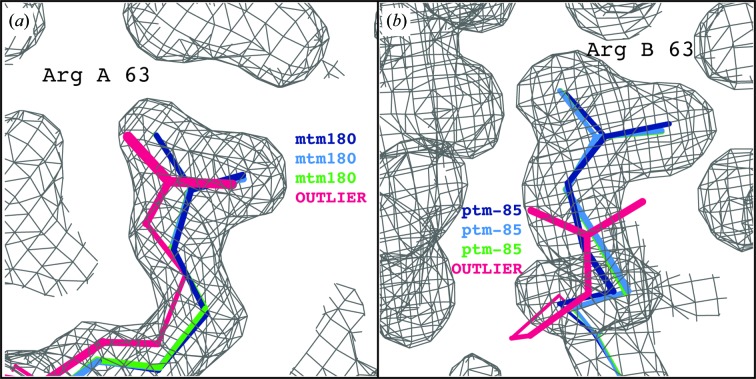
Comparison of Arg63 of PDB entry 1sar following molecular replacement plus *AutoBuild* with refinement in *phenix.refine*. (*a*) Arg63 from the *A* chain. The final position is shown for 1sar (dark blue), refinement with torsion NCS restraints (light blue), refinement without NCS restraints (green) and refinement with global NCS restraints (hot pink), with rotamer states indicated in matching colors. (*b*) Arg63 from the *A* chain. The final position is shown for PDB entry 1sar (dark blue), refinement with torsion NCS restraints (light blue), refinement without NCS restraints (green) and refinement with global NCS restraints (hot pink), with rotamer states indicated in matching colors. All maps are 2*mF*_o_ − *DF*_c_ maps contoured at 1σ. Images were generated using *KiNG* (Chen *et al.*, 2009 ▶).

*AutoBuild* (Terwilliger, 2002 ▶; Terwilliger *et al.*, 2008 ▶) model rebuilding was then performed using three different protocols: no NCS in model refinement, global (Cartesian-based) NCS as part of model refinement and torsion NCS with rotamer correction and consistency checks as part of model refinement. Ordered water picking was disabled, and default settings were used otherwise. Using the traditional global NCS target, both Arg63 side chains are distorted to outlier conformations, while the flexible torsion-based NCS target allows these residues to reach and maintain valid rotamer states automatically (Fig. 3 ▶). Validation statistics are summarized in Table 1 ▶. Running *AutoBuild* without NCS and with torsion NCS produces final models with similar *R* factors, with the torsion NCS model having a slightly smaller *R*_free_–*R*_work_ gap (Brünger, 1992 ▶), consistent with a less overfitted model. By comparison, the global NCS model has much higher *R* factors. There is one fewer rotamer outlier in the torsion NCS refined model, consistent with rotamer correction as part of the torsion NCS method. Compared with the deposited structure, the full-atom r.m.s.d.s for each *AutoBuild* model are 0.543, 0.508 and 0.625 Å for no NCS, torsion NCS and global NCS, respectively. Full-atom r.m.s.d.s were calculated using *VMD* (Humphrey *et al.*, 1996 ▶). The clashscore is slightly elevated when using either NCS implementation: 1.74 *versus* 1.39 when using no NCS restraints. Visual inspection reveals that the difference is a single clash between the CG atom of Arg*A*40 and the HA2 atom of Gly*B*61. These atoms clash to some degree in all three refinements, but the refinement with no NCS restraints produces a model in which the overlap is just below the cutoff of 0.4 Å, resulting in the lower clashscore.

**Figure 4:**
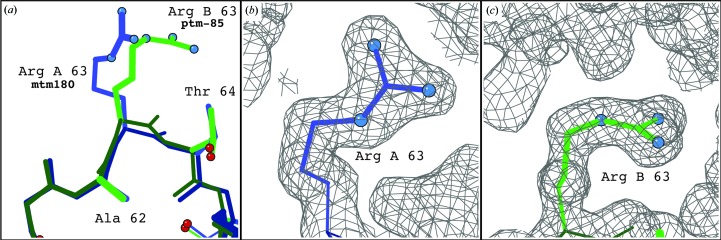
Comparison of the Arg63 loop in the *A* and *B* chains in the 1.8 Å resolution RNAse S structure (PDB entry 1sar). (*a*) Overlay of the Arg63 loop region from the *A* chain (blue) and *B* chain (green), illustrating the rotameric difference of Arg63 between the chains. (*b*) Arg63 from the *A* chain with 2*mF*_o_ − *DF*_c _density map. (*c*) Arg63 from chain *B* with 2*mF*_o_ − *DF*_c_ density map. All maps are contoured at 1σ. Images were generated using *KiNG* (Chen *et al.*, 2009 ▶).

The two NCS-related chains in the asymmetric unit exhibit conformational differences supported by the density, particularly in the loop region surrounding Arg63. As seen in Fig. 4 ▶(*a*), Arg*A*63 is best fitted as an **mtm180** rotamer, while Arg*B*63 is best fitted as a **ptm−180** rotamer (Fig. 4 ▶ *b*), which is consistent with the rotamers observed in the deposited 1sar structure.

### 2.4. Testing torsion NCS restraints in a typical MR workflow at moderate resolution   

**Figure 5:**
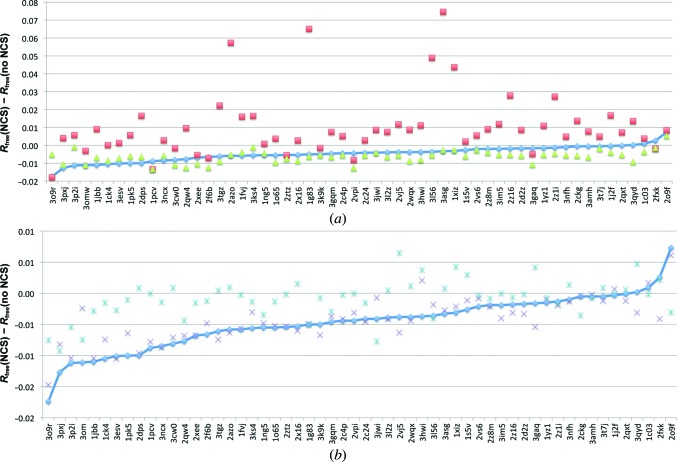
(*a*) Plot of residual *R*_free_ values for refinements of a set of 56 moderate-resolution structures using torsion NCS with rotamer correction (blue diamonds), global NCS (red squares) and local NCS restraints in *REFMAC*5 (Murshudov *et al.*, 2011 ▶; green squares). The residual *R*_free_ is calculated as *R*_free_(NCS) − *R*_free_(no NCS). (*b*) Plot of residual *R*_free_ values for refinements using torsion NCS restraints only (purple crosses), rotamer correction only (light blue dashed crosses) and torsion NCS with rotamer correction (blue diamonds). Data for both plots are plotted on the *x* axis in order of increasing residual *R*_free_ for refinement with torsion NCS with rotamer correction in *phenix.refine*.

To test the effectiveness of torsion-based NCS restraints in a typical molecular replacement-driven workflow, we randomly selected 56 protein structures from the PDB between 2.0 and 3.0 Å resolution which have between two and four NCS copies, no ligands and are between 100 and 300 amino acids in length. This data set is summarized in Supplementary Table S1^1^. We also required each structure to have a homologue with sequence similarity of >90% but <100% for use for molecular replacement. We required this high level of similarity to limit the need for any manual rebuilding, allowing us to test the effectiveness of torsion NCS restraints in a fully automated mode of operation within *Phenix*. Once phased using molecular replacement in *Phaser* (McCoy *et al.*, 2007 ▶), MR solutions were placed in the same frame of reference as the deposited PDB entry using *phenix.alt_orig_sym_mate* (Oeffner *et al.*, 2012 ▶), and were then processed with *AutoBuild* (Terwilliger, 2002 ▶; Terwilliger *et al.*, 2008 ▶) using the rebuild-in-place option with no NCS in refinement and no placing of waters. Following *AutoBuild*, models were refined using *phenix.refine* for ten macro-cycles, refining individual sites and individual ADPs, and optimizing target weights for *xyz* sites. Each refinement was repeated with no NCS, global NCS and torsion NCS restraints. As shown in Fig. 5 ▶(*a*), the use of torsion NCS restraints and rotamer correction generally results in the same or a lower *R*_free_ value when compared with using no NCS restraints. By comparison, global NCS restraints often result in much larger values of *R*_free_, often coupled with significant distortions of the model. In a handful of cases the global NCS restraints result in a slightly lower *R*_free_ value, but visual examination of these models reveal no significant structural differences between these models and those refined with torsion NCS restraints. We chose to report residual *R*_free_ values, *R*_free_(NCS) − *R*_free_(no NCS), rather than absolute *R*_free_ values because at this early stage of refinement the trend in *R*_free_ is more revealing than its absolute magnitude. Refinements would need to be completed, including building the handful of missing side chains and placing any ordered solvent and/or ions, for comparison with published *R*_free_ values to be revealing.

To test the relative contribution of the torsion NCS term and rotamer correction, we also ran these refinements with torsion NCS restraints alone and rotamer correction alone. As shown in Fig. 5 ▶(*b*), rotamer correction alone generally results in *R*_free_ values greater than or equal to the combined approach, with 18/56 cases (∼32%) resulting in a worse *R*_free_ than using no NCS restraints at all. By comparison, using torsion NCS restraints alone produces results that correlate more closely with the combined approach, with only 4/56 cases (∼7%) resulting in an *R*_free_ value worse than using no NCS at all. Using both torsion NCS restraints and rotamer correction combined results in the most consistent results across this data set.

These refinement results were also compared with refinements carried out using *REFMAC*5 (Murshudov *et al.*, 2011 ▶). We ran *REFMAC*5 both with and without local NCS restraints to allow us to calculate internally consistent *R*_free_(NCS) − *R*_free_(no NCS) values. As shown in Fig. 5 ▶(*a*), refinement in *REFMAC*5 using local NCS restraints exhibits the same trend of improvement in *R*_free_ over refinement in *REFMAC*5 without local NCS restraints as observed for the *phenix.refine* results. On average, the addition of local NCS restraints in *REFMAC*5 reduces *R*_free_ by −0.62%, while torsion NCS restraints with rotamer correction in *phenix.refine* reduces *R*_free_ by −0.47%. Both methods at worst produce the same *R*_free_ as refinement without NCS restraints but for a handful of cases (PDB entries 1c03, 2fxk and 2o9f for torsion NCS with rotamer correction, 2o9f for *REFMAC*5). These results suggest that both NCS parameterizations are a suitable automated strategy for the moderate-resolution cases presented in this test set.

**Figure 6:**
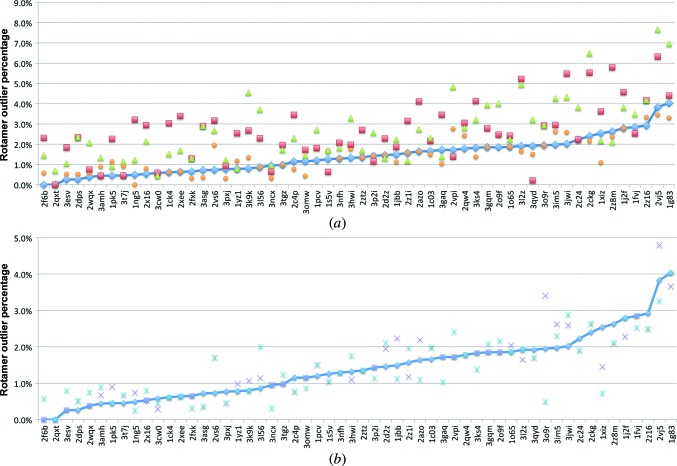
(*a*) Rotamer outlier percentage analysis for a set of 56 test refinements using torsion NCS with rotamer correction (blue diamonds), global NCS (red triangles), no NCS (orange circles) and *REFMAC*5 (green triangles). (*b*) Rotamer outlier percentage for torsion NCS restraints only (purple crosses), rotamer correction only (light blue dashed crosses) and torsion NCS with rotamer correction (blue diamonds). Both plots are sorted by increasing rotamer outlier percentage using torsion NCS restraints with rotamer correction.

Geometric validation metrics demonstrate similar results. As shown in Fig. 6 ▶(*a*), the rotamer outlier percentage from refinements using torsion NCS restraints with rotamer correction is similar to those from refinements with no NCS restraints (average of 1.41 and 1.43%, respectively), with many cases of a higher rotamer outlier percentage when using global NCS restraints (average of 2.62%) or *REFMAC*5 (average of 2.64%). As shown in Fig. 6 ▶(*b*), torsion NCS restraints alone and rotamer correction alone exhibit a similar trend to that of the combined approach, with torsion NCS alone having an average outlier percentage of 1.48% and rotamer correction alone having an average outlier percentage of 1.39%.

**Figure 7:**
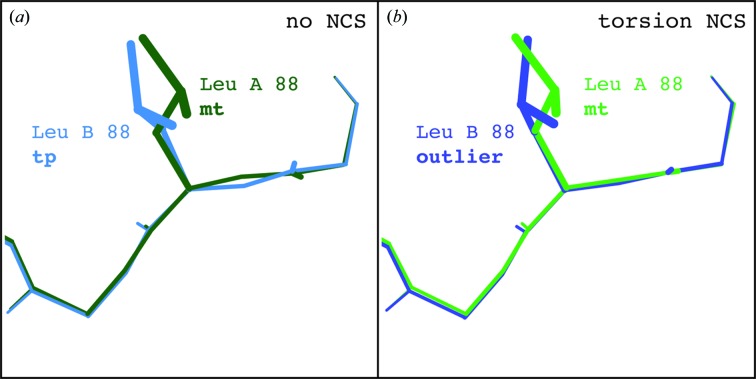
Comparison of NCS-related Leu*A*88 and Leu*B*88 of PDB entry 1jbb refined with and without torsion NCS restraints. (*a*) Refinement without NCS restraints allows the incorrectly built Leu*B*88 side chain to remain a **tp** rotamer while distorting the surrounding backbone geometry. (*b*) Refinement using torsion NCS restraints preserves similar backbone geometry, which causes the incorrectly built Leu*B*88 side chain to present as an outlier. Images were generated using *KiNG* (Chen *et al.*, 2009 ▶).

Ramachandran analysis produces similar results. As shown in Fig. 7 ▶(*a*), refinement with no NCS, torsion NCS with rotamer correction or refinement with *REFMAC*5 all produce similar percentages of Ramachandran outliers, with averages of 0.46, 0.42 and 0.52%, respectively. The results from the refinements using global NCS restraints are slightly worse, with an average Ramachandran outlier percentage of 0.59%. Fig. 7 ▶(*b*) illustrates that refinements with torsion NCS alone produce models with slightly better average Ramachandran outlier percentages than refinements with rotamer correction alone (0.39 *versus* 0.47%) but, like rotamer outlier percentages, the trend is similar.

**Figure 8:**
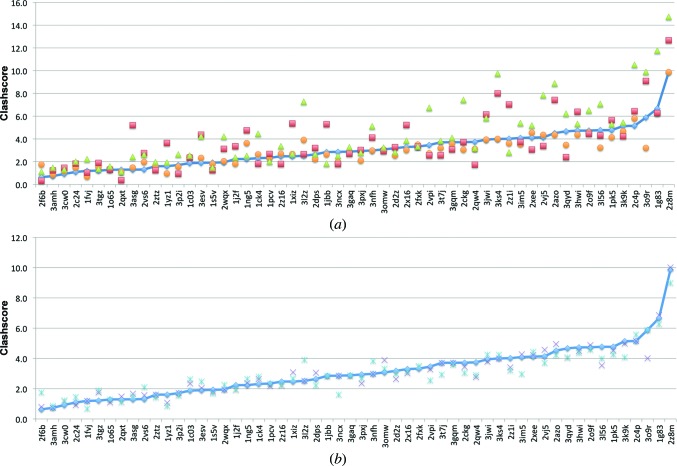
(*a*) Clashscore (Chen *et al.*, 2010 ▶) analysis for a set of 56 test refinements using torsion NCS with rotamer correction (blue diamonds), global NCS (red triangles), no NCS (orange circles) and *REFMAC*5 (green triangles). (*b*) Clashscore analysis for torsion NCS restraints only (purple crosses), rotamer correction only (light blue dashed crosses) and torsion NCS with rotamer correction (blue diamonds). Both plots are sorted by increasing rotamer outlier percentage using torsion NCS restraints with rotamer correction.

As shown in Fig. 8 ▶(*a*), refinement using torsion NCS restraints with rotamer correction produces slightly elevated clashscores compared with refinement using no NCS restraints (averages of 3.10 and 2.97, respectively), while refinement using global NCS restraints causes elevated clashscores in many cases (average clashscore of 3.67). Refinements with *REFMAC*5 produce the highest clashscores across the test set, with an average of 4.39. Rotamer correction alone and torsion NCS restraints alone produce similar results to the combined approach (average clashscores of 3.01 and 3.05, respectively), with the slightly better performance by rotamer correction alone likely to be owing to an increased emphasis on not introducing steric clashes, coupled with fewer restraints on the overall model. As described in Chen *et al.* (2010 ▶), clashscore is defined as the number of steric clashes >0.4 Å per 1000 atoms. These differences in clashscore, therefore, are minimal, but serve to show that in general the use of torsion NCS restraints results in a model approximately as good as, if not better than, those models refined with no NCS restraints, and are usually safe to use at this working resolution range, even very early in the refinement process.

**Figure 9:**
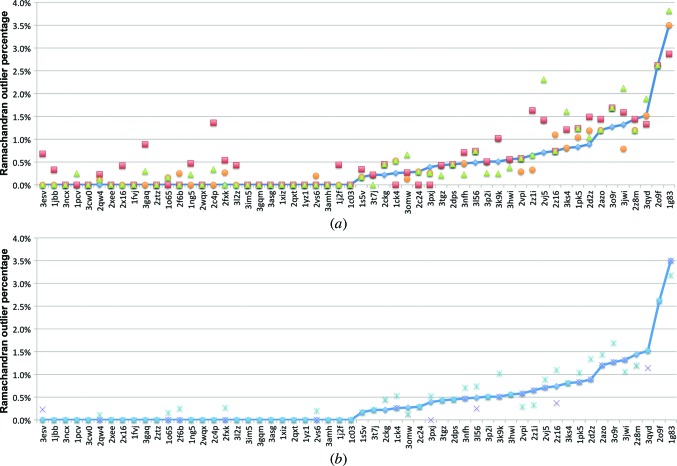
(*a*) Ramachandran outlier percentage analysis for a set of 56 test refinements using torsion NCS with rotamer correction (blue diamonds), global NCS (red triangles), no NCS (orange circles) and *REFMAC*5 (green triangles). (*b*) Ramachandran outlier percentage for torsion NCS restraints only (purple crosses), rotamer correction only (light blue dashed crosses) and torsion NCS with rotamer correction (blue diamonds). Both plots are sorted by increasing Ramachandran outlier percentage using torsion NCS restraints with rotamer correction.

Interestingly, as shown in Fig. 5 ▶(*b*), of the three cases in which torsion NCS with rotamer correction results in higher *R*_free_ values than with no NCS restraints at all, rotamer correction alone corrects this problem in two cases (PDB entries 1c03 and 2o9f) and torsion NCS restraints alone corrects this problem in the other case (PDB entry 2fxk). Closer inspection of 1c03 reveals that the model has perfect Ramachandran statistics for all refinements (Fig. 9 ▶), limiting the benefit of torsion NCS restraints on the backbone. The refinement without any NCS restraints produces the lowest rotamer outlier percentage for this example, suggesting that rotamer correction is too aggressive in this case and, combined with torsion NCS restraints, produces a poorer model. For 2fxk, an improvement in rotamer outlier percentage with rotamer correction comes at the cost of an increase in the number of Ramachandran outliers, leading to an overfitted model, explaining the overall improvement for the torsion NCS only refinement. Finally, for 2o9f, all models fall into the bottom third of each geometry validation metric (third from last in the Ramachandran outlier percentage), suggesting that the refinement of models that are quite far from the correct global minimum requires more concerted motions than are possible with the addition of simple restraints, and that the addition of too many restraints further limits the ability of the model to move towards this minimum.

**Figure 10:**
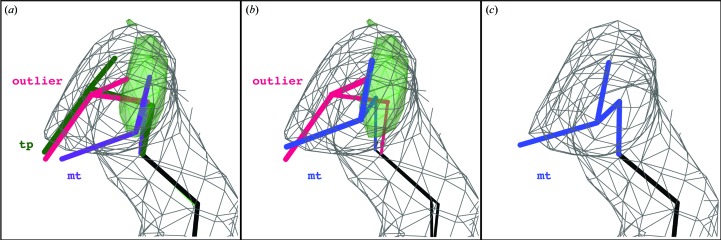
Handling of Leu*B*88 in the refinement of PDB entry 1jbb (2.0 A resolution). (*a*) Following ten macro-cycles of *phenix.refine*, Leu*B*88 refines to an incorrect **tp** rotamer (no NCS restraints, shown in green) or an outlier (torsion NCS restraints, shown in pink). Simple rotation to a correct **mt** rotamer (purple) does not improve the density fit sufficiently for acceptance by the rotamer correction routine. (*b*) Correct placement of the **mt** rotamer (blue) following ‘autofit best rotamer’ using *Coot* (Emsley *et al.*, 2010 ▶). (*c*) Following five additional macro-cycles of *phenix.refine* using torsion NCS restraints, the correct **mt** rotamer remains and the positive *mF*_o_ − *DF*_c_ density peak is eliminated. 2*mF*_o_ − *DF*_c_ maps (gray mesh) are contoured at 1.2σ. *mF*_o_ − *DF*_c_ maps (green peak) are contoured at 3.5σ. Images were generated using *KiNG* (Chen *et al.*, 2009 ▶).

On occasion, the final model following refinement using torsion NCS restraints will have a slightly higher rotamer outlier percentage or clashscore than a comparable model refined using no NCS restraints. In our experience, this almost always indicates an area of the model that requires concerted rebuilding beyond the capacity of current automated refinement methods. For example, from the 56 models selected for this test, the final torsion NCS-refined model for the 2.0 Å resolution ubiquitin-conjugating enzyme structure (PDB entry 1jbb; VanDemark *et al.*, 2001 ▶) has a rotamer outlier percentage of 1.49% (a total of four outliers) *versus* a default-refined model outlier percentage of 1.12% (a total of three outliers), coupled with an elevated clashscore (2.85 *versus* 2.65). The difference is an outlier for Leu*B*88 using torsion NCS restraints *versus* a **tp** rotamer when using no NCS restraints. While the Leu*A*88 side chain is an **mt** rotamer, the model around the side chain is too distorted for either the rotamer outlier or rotamer consistency routines to correct this change. As shown in Fig. 7 ▶, however, the use of torsion NCS restraints is able to refine to similar backbone orientations between the *A* and *B* chain, causing the incorrect side chain to stand out as an outlier. Using no NCS restraints, this side chain distributes the error across the local backbone, refining to a false-positive **tp** rotamer (Figs. 7 ▶ *a* and 10 ▶ *a*). The clashscore is also eased by distributing the error across the backbone, explaining the higher observed clashscore with torsion NCS restraints. The ϕ/ψ values around Leu*A*88 (−138.5°, 140.3°) are quite different from those around Leu*B*88 (−155.5°, 125.9°) when refined with no NCS. Conversely, the ϕ/ψ values are quite similar when refined using flexible torsion NCS restraints [(−145.5°, 134.3°) and (−147.9°, 130.7°)]. Outliers such as these can be corrected using more aggressive refinement methods or through simple rebuilding in a graphical building program such as *Coot* (Emsley *et al.*, 2010 ▶). In this case, the side chain is corrected to an **mt** rotamer using *Coot* (Fig. 10 ▶ *b*), and subsequent refinement confirms that this is a preferable rotamer for this side chain (Fig. 10 ▶ *c*).

Following five additional macro-cycles of refinement, the torsion NCS-refined model with corrected Leu*B*88 has improved *R*_work_/*R*_free_ values (0.1942/0.2441) compared with the model with corrected Leu*B*88 refined with no NCS restraints (0.2015/0.2503). The final rotamer outlier percentages favor the torsion NCS-refined model (1.12 *versus* 1.49%).

### 2.5. Testing re-refinement at a wide range of resolutions   

To test the safety and efficacy of using torsion NCS restraints at a wide range of resolutions, we selected a set of deposited PDB structures ranging from 1.0 to 4.1 Å resolution roughly in increments of 0.1 Å. Structures were chosen that had between two and six NCS copies, usable structure factors and *R*_free_ sets, and no ligands. This data set is summarized in Supplementary Table S2. Each structure was re-refined using *phenix.refine* for five macro-cycles, refining individual sites (with weight optimization) and individual ADPs. The results are summarized in Supplementary Fig. S1.

The expectation in re-refining deposited models is that they are already well re-refined, and it is unlikely that simple refinement will greatly change the model or validation statistics. As shown in Supplementary Fig. S1(*a*), *R*_free_ values generally improved slightly upon re-refinement (compared with the value for the deposited model), with the exception being significant increases when using global NCS restraints in some cases (PDB entries 3d95, 4dov, 4ilj and 2vr9). The average change in *R*_free_ using no NCS parameterization is −0.007, with the largest decrease in *R*_free_ being −0.047 for PDB entry 1xdv (4.1 Å) and the largest increase in *R*_free_ being +0.024 for PDB entry 4i6p (2.9 Å). By comparison, the use of global NCS restraints often results in an increased *R*_free_ compared with no NCS restraints, with an average increase of +0.016. The use of torsion NCS restraints alone resulted in an average decrease in *R*_free_ of −0.002, while the use of NCS-related rotamer correction resulted in an average decrease of −0.001. When combined together, torsion NCS restraints with rotamer correction results in an average decrease of −0.003. These changes, while subtle, support the claim that the use of torsion NCS restraints, particularly in conjunction with NCS-related rotamer correction, are safe to use across a wide resolution range, resulting in models that are similar or slightly better than those derived from refinement with no NCS restraints.

To further validate these results, we also looked at rotamer outlier percentage (Supplementary Fig. S1*b*), Ramachandran outlier percentage (Supplementary Fig. S1*c*) and clashscore (Supplementary Fig. S1*d*). Compared with the PDB-deposited models, refinement with *phenix.refine* with no NCS results in an average decrease in the rotamer outlier percentage of −2.34%, while refinement with global NCS results in an average decrease of only −0.24%. Refinement with torsion NCS alone, NCS-related rotamer correction alone and torsion NCS combined with rotamer correction results in average decreases of −2.41, −2.54 and −2.54%, respectively, when compared with the PDB-deposited models.

Ramachandran analysis of these results demonstrates that refinement with no NCS results in an average change in Ramachandran outlier percentage of −1.13% when compared with the deposited models in the PDB, while refinement with global NCS restraints results in an average change of −0.63%. Refinement with torsion NCS alone, NCS-related rotamer correction alone and torsion NCS combined with rotamer correction results in average changes of −1.12, −1.15 and −1.10%, respectively, when compared with the PDB-deposited models.

Clashscore analysis of these results demonstrates that refinement with no NCS results in an average change in clashscore of −12.44 when compared with the deposited PDB models, while refinement with global NCS restraints results in an average change of −9.63. Refinement with torsion NCS alone, NCS-related rotamer correction alone and torsion NCS combined with rotamer correction result in average changes in clashscore of −11.98, −12.25 and −11.89, respectively, when compared with the PDB-deposited model.

Overall, these validation results are consistent with our observation that refinement with torsion NCS restraints with rotamer correction produces models as good as or better than those produced by refinement with no NCS. Refinement with global NCS, on the other hand, is not as successful in improving the PDB-deposited models, producing models with worse validation statistics than refinement with no NCS restraints or with torsion NCS with rotamer correction.

## 3. Discussion   

In this manuscript, we introduce flexible torsion-based NCS restraints for refinement of crystallographic structures of macromolecules. We select torsion space as a parameterization space for these restraints, as torsion angles have a strong correlation to well characterized structural features such as amino-acid side-chain rotamers and RNA backbone conformations. Further, using torsion angles allows a minimal set of restraints to fully describe the in-sequence NCS relationship. By comparison, methods that use interatomic distances to restrain local NCS relationships do so by capturing longer range spatial relationships between NCS-related copies, but are likely to require the addition of more restraints to achieve the same coverage of fold-space as achieved with our torsion-based approach. Further, distance-based restraints do not directly relate to torsion-based folding expectations, such as side-chain rotamers and backbone conformations in proteins. As a result, rotameric errors may be preserved or introduced by restraining local distances, which is supported by the higher rotamer outlier percentages observed in the refinements carried out using *REFMAC*5 with local NCS restraints carried out in this study.

By using a flexible target function, we allow local differences between NCS-related chains while maintaining a fully automated functionality. This parameterization avoids the need for any manual definition of NCS groups and is independent of the Euclidean relationship between related chains. Rotamer correction and consistency algorithms allow automated correction of modeling errors in many cases, reducing the need for manual rebuilding and decreasing the necessary number of total refinement macro-cycles. Further, as shown in the example of Leu88 in PDB entry 1jbb, the use of torsion NCS restraints at early stages of refinement can cause incorrectly built side chains to remain as outliers even after several rounds of refinement, allowing identification and correction early in the refinement process. Clashscores, in particular, are observed to be elevated when using NCS restraints, particularly in cases where there are still a significant percentage of uncorrected rotamer outliers, where the increased rigidity introduced through torsion restraints prevents these errors from being distributed across the backbone. The positive aspect of concentrating these errors is that it simplifies the identification of problem areas, directing the crystallographer to the areas of a model that need the most manual rebuilding.

One outstanding limitation for both torsion-based and global, Cartesian-based, NCS approaches is that in order for true differences between NCS-related copies to be properly refined differences must be introduced in the model prior to refinement. If NCS-related models are exactly the same, then the NCS contribution to the geometry target will be zero, which is a minimum that simple refinement is unlikely to escape. This situation commonly arises in the case of molecular replacement where the same search model is used to fit all chains. Some method of randomization, whether it be minimization, simulated annealing or automated model building (such as *AutoBuild*), typically needs to be employed as part of the initial modeling process for NCS restraints to be used and still allow conformational differences between related chains. Thus, we recommend that models containing NCS-related chains which are solved by molecular replacement at sufficiently high resolution be rebuilt with *AutoBuild* with NCS refinement disabled to allow initial differences to be introduced prior to application of an appropriate refinement strategy for the working resolution. In the future, methods that combine refinement and local rebuilding could provide an alternative approach to automatically breaking NCS.

## Supplementary Material

Supporting Information.. DOI: 10.1107/S1399004714003277/rr5054sup1.pdf

## Figures and Tables

**Table 1:** *AutoBuild* results for PDB entry 1sar

	*R*	*R*_free_	*R* gap	Rotamer outliers (%)	Ramachandran favored (%)	Clashscore
No NCS	0.2170	0.2537	0.0367	1.22	100	1.39
Global NCS	0.2385	0.2703	0.0318	3.66	100	1.74
Torsion NCS	0.2174	0.2501	0.0327	0.61	100	1.74

## Footnotes

1 Supporting information has been deposited in the IUCr electronic archive (Reference: RR5054).

## References

[bb1] Afonine, P. V., Grosse-Kunstleve, R. W., Echols, N., Headd, J. J., Moriarty, N. W., Mustyakimov, M., Terwilliger, T. C., Urzhumtsev, A., Zwart, P. H. & Adams, P. D. (2012). *Acta Cryst.* D**68**, 352–367. 10.1107/S0907444912001308 PMC3322595 22505256

[bb2] Bricogne, G. (1976). *Acta Cryst.* A**32**, 832–847.

[bb3] Bricogne, G., Blanc, E., Brandl, M., Flensburg, C., Keller, P., Paciorek, W., Roversi, P., Sharff, A., Smart, O. & Vonrhein, C. (2010). *BUSTER.* Global Phasing Limited, Cambridge, England.

[bb4] Brünger, A. T. (1992). *Nature (London)*, **355**, 472–475. 10.1038/355472a0 18481394

[bb5] Brünger, A. T., Adams, P. D., Clore, G. M., DeLano, W. L., Gros, P., Grosse-Kunstleve, R. W., Jiang, J.-S., Kuszewski, J., Nilges, M., Pannu, N. S., Read, R. J., Rice, L. M., Simonson, T. & Warren, G. L. (1998). *Acta Cryst.* D**54**, 905–921. 10.1107/s0907444998003254 9757107

[bb6] Burnley, B. T., Afonine, P. V., Adams, P. D. & Gros, P. (2012). *Elife*, **1**, e00311. 10.7554/eLife.00311 PMC3524795 23251785

[bb7] Chen, V. B., Arendall, W. B., Headd, J. J., Keedy, D. A., Immormino, R. M., Kapral, G. J., Murray, L. W., Richardson, J. S. & Richardson, D. C. (2010). *Acta Cryst.* D**66**, 12–21. 10.1107/S0907444909042073 PMC2803126 20057044

[bb8] Chen, V. B., Davis, I. W. & Richardson, D. C. (2009). *Protein Sci.* **18**, 2403–2409. 10.1002/pro.250 PMC2788294 19768809

[bb9] Davis, I. W., Arendall, W. B., Richardson, D. C. & Richardson, J. S. (2006). *Structure*, **14**, 265–274. 10.1016/j.str.2005.10.007 16472746

[bb10] Dennis, J. E. Jr & Welsch, R. E. (1978). *Commun. Statist. Simulation Comput.* **7**, 345–359.

[bb11] Emsley, P., Lohkamp, B., Scott, W. G. & Cowtan, K. (2010). *Acta Cryst.* D**66**, 486–501. 10.1107/S0907444910007493 PMC2852313 20383002

[bb12] Georgiev, I., Keedy, D., Richardson, J. S., Richardson, D. C. & Donald, B. R. (2008). *Bioinformatics*, **24**, i196–i204. 10.1093/bioinformatics/btn169 PMC2718647 18586714

[bb13] Gildea, R. J., Bourhis, L. J., Dolomanov, O. V., Grosse-Kunstleve, R. W., Puschmann, H., Adams, P. D. & Howard, J. A. K. (2011). *J. Appl. Cryst.* **44**, 1259–1263. 10.1107/S0021889811041161 PMC3236671 22199401

[bb14] Headd, J. J., Echols, N., Afonine, P. V., Grosse-Kunstleve, R. W., Chen, V. B., Moriarty, N. W., Richardson, D. C., Richardson, J. S. & Adams, P. D. (2012). *Acta Cryst.* D**68**, 381–390. 10.1107/S0907444911047834 PMC3322597 22505258

[bb15] Headd, J. J., Immormino, R. M., Keedy, D. A., Emsley, P., Richardson, D. C. & Richardson, J. S. (2009). *J. Struct. Funct. Genomics*, **10**, 83–93. 10.1007/s10969-008-9045-8 PMC2704614 19002604

[bb16] Hendrickson, W. A. (1985). *Methods Enzymol.* **115**, 252–270. 10.1016/0076-6879(85)15021-4 3841182

[bb17] Humphrey, W., Dalke, A. & Schulten, K. (1996). *J. Mol. Graph.* **14**, 33–38. 10.1016/0263-7855(96)00018-5 8744570

[bb18] Keedy, D. A., Georgiev, I., Triplett, E. B., Donald, B. R., Richardson, D. C. & Richardson, J. S. (2012). *PLoS Comput. Biol.* **8**, e1002629. 10.1371/journal.pcbi.1002629 PMC3410847 22876172

[bb19] Kleywegt, G. J. (1996). *Acta Cryst.* D**52**, 842–857. 10.1107/S0907444995016477 15299650

[bb20] Lovell, S. C., Word, J. M., Richardson, J. S. & Richardson, D. C. (2000). *Proteins*, **40**, 389–408. 10861930

[bb21] Lovell, S. C., Davis, I. W., Arendall, W. B. III, de Bakker, P. I., Word, J. M., Prisant, M. G., Richardson, J. S. & Richardson, D. C. (2003). *Proteins*, **50**, 437–450. 10.1002/prot.10286 12557186

[bb22] McCoy, A. J., Grosse-Kunstleve, R. W., Adams, P. D., Winn, M. D., Storoni, L. C. & Read, R. J. (2007). *J. Appl. Cryst.* **40**, 658–674. 10.1107/S0021889807021206 PMC2483472 19461840

[bb23] Murshudov, G. N., Skubák, P., Lebedev, A. A., Pannu, N. S., Steiner, R. A., Nicholls, R. A., Winn, M. D., Long, F. & Vagin, A. A. (2011). *Acta Cryst.* D**67**, 355–367. 10.1107/S0907444911001314 PMC3069751 21460454

[bb24] Nicholls, R. A., Long, F. & Murshudov, G. N. (2012). *Acta Cryst.* D**68**, 404–417. 10.1107/S090744491105606X PMC3322599 22505260

[bb25] Oeffner, R., Bunkoczi, G. & Read, R. J. (2012). *Comput. Crystallogr. Newsl.* **3**, 5–10.

[bb26] Ramachandran, G. N. & Sasisekharan, V. (1968). *Adv. Protein Chem.* **23**, 283–438. 10.1016/s0065-3233(08)60402-7 4882249

[bb27] Richardson, J. S., Schneider, B., Murray, L. W., Kapral, G. J., Immormino, R. M., Headd, J. J., Richardson, D. C., Ham, D., Hershkovits, E., Williams, L. D., Keating, K. S., Pyle, A. M., Micallef, D., Westbrook, J. & Berman, H. M. (2008). *RNA*, **14**, 465–481. 10.1261/rna.657708 PMC2248255 18192612

[bb28] Rossmann, M. G. (1972). *The Molecular Replacement Method.* New York: Gordon & Breach.

[bb29] Sevcik, J., Dodson, E. J. & Dodson, G. G. (1991). *Acta Cryst.* B**47**, 240–253. 1654932

[bb30] Sevcik, J., Zegers, I., Wyns, L., Dauter, Z. & Wilson, K. S. (2005). *Eur. J. Biochem.* **216**, 301–305. 10.1111/j.1432-1033.1993.tb18145.x 8396032

[bb31] Sheldrick, G. M. (2008). *Acta Cryst.* A**64**, 112–122. 10.1107/S0108767307043930 18156677

[bb32] Smart, O., Brandl, M., Flensburg, C., Keller, P., Paciorek, W., Vonrhein, C., Womack, T. & Bricogne, G. (2008). *Abstr. Annu. Meet. Am. Crystallogr. Assoc.*, Abstract TP139, p. 117.

[bb33] Smart, O. S., Womack, T. O., Flensburg, C., Keller, P., Paciorek, W., Sharff, A., Vonrhein, C. & Bricogne, G. (2012). *Acta Cryst.* D**68**, 368–380. 10.1107/S0907444911056058 PMC3322596 22505257

[bb34] Symersky, J., Li, S., Carson, M. & Luo, M. (2003). *Proteins*, **51**, 484–486. 10.1002/prot.10364 12696058

[bb35] Terwilliger, T. C. (2002). *Acta Cryst.* D**58**, 1937–1940. 10.1107/s0907444902016438 12393925

[bb36] Terwilliger, T. C., Grosse-Kunstleve, R. W., Afonine, P. V., Moriarty, N. W., Zwart, P. H., Hung, L.-W., Read, R. J. & Adams, P. D. (2008). *Acta Cryst.* D**64**, 61–69. 10.1107/S090744490705024X PMC2394820 18094468

[bb37] Tronrud, D. E., Ten Eyck, L. F. & Matthews, B. W. (1987). *Acta Cryst.* A**43**, 489–501.

[bb38] Urzhumtsev, A. G., Lunin, V. Yu. & Vernoslova, E. A. (1989). *J. Appl. Cryst.* **22**, 500–506.

[bb39] Usón, I., Pohl, E., Schneider, T. R., Dauter, Z., Schmidt, A., Fritz, H. J. & Sheldrick, G. M. (1999). *Acta Cryst.* D**55**, 1158–1167. 10.1107/s0907444999003972 10329778

[bb40] VanDemark, A. P., Hofmann, R. M., Tsui, C., Pickart, C. M. & Wolberger, C. (2001). *Cell*, **105**, 711–720. 10.1016/s0092-8674(01)00387-7 11440714

[bb41] Westbrook, J. D. & Fitzgerald, P. M. D. (2005). *Structural Bioinformatics*, edited by P. E. Bourne & H. Weissig, pp. 161–180. Hoboken: John Wiley & Sons.

