# Supplementary material for: Flexible torsion-angle noncrystallographic symmetry restraints for improved macromolecular structure refinement
Source: Acta Crystallogr D Biol Crystallogr. 2014 Apr 30;70(Pt 5):1346–56. doi: 10.1107/S1399004714003277 (PMC4014122; doi:10.1107/S1399004714003277)
Supplement: Supplementary file 1 [file d-70-01346-sup1.pdf]

Acta Crystallographica Section D

Volume 70 (2014)

Supporting information for article:

Flexible torsion angle non-crystallographic symmetry restraints for improved macromolecular structure refinement

Jeffrey J. Headd, Nathaniel Echols, Pavel V. Afonine, Nigel W. Moriarty, Richard J. Gildea and Paul D. Adams

Table S1 Moderate Resolution MR Test Set

PDB ID	Resolution	# NCS copies	MR search model PDB ID
1c03	2.30	4	1c02
1ck4	2.20	2	1pt6
1fvj	2.06	2	1dsb
1g83	2.60	2	3uf4
1j2f	2.30	2	3a77
1jbb	2.00	2	2gmi
1ng5	2.00	2	1qwz
1o65	2.33	3	1o67
1pcv	2.30	2	2i0w
1pk5	2.40	2	1zh7
1s5v	2.35	2	3i7q
1xiz	2.00	2	3lf6
1yz1	2.00	4	3ebm
2azo	2.30	2	1azo
2c24	2.27	2	1wmx
2c4p	2.35	2	2yho
2ckg	2.45	2	2xph
2d2z	2.20	3	2ahe
2dps	2.40	2	2cxa
2f6b	2.80	2	1qh6
2fxk	2.54	2	1yd9
2o9f	2.55	2	1rc2
2qw4	2.80	4	1yje
2qxt	2.00	2	1t4m
2vj5	3.00	2	2vix
2vpi	2.40	2	2vxo
2vs6	2.40	2	2jdl
2wqx	2.03	2	2uzx
2x16	2.13	2	2vei
2xee	2.10	4	2xeh
2z16	2.02	2	3md2
2z1i	2.00	2	2z1g
2z8m	2.00	2	2q8y
2ztz	2.00	2	3zye
3amh	2.09	2	3amn
3asg	2.33	2	3as4

3cw0	2.40	4	3efp
3esv	2.00	2	3et9
3gaq	2.25	2	1qft
3gqm	2.10	2	3eir
3hwi	2.29	2	3aay
3im5	2.55	2	3im6
3jwi	2.20	2	3jwg
3k9k	2.55	2	3k9j
3ks4	2.40	2	3l2a
3l2z	2.80	2	2cgh
3l56	2.30	2	3cw4
3ncx	2.60	2	2zqk
3nfh	2.17	2	3nfi
3o9r	2.00	2	3l4q
3omw	2.87	4	3p9y
3p2i	2.40	2	3pb3
3pxj	2.30	4	2yd1
3qyd	2.97	3	3i2x
3t7j	2.04	2	3t7i
3tgz	2.30	2	3p90

Table S2 1.0Å-4.1Å Re-refinement Test Set

PDB ID	Resolution	# NCS Copies
1mn8	1.00	4
3m9j	1.10	2
3d95	1.20	2
1kmt	1.30	2
2ohw	1.40	2
3w3e	1.50	2
4j7w	1.60	2
4dov	1.70	2
4guz	1.80	4
3v3s	1.90	2
4ilj	2.00	2
4gkf	2.10	2
4lq8	2.20	2
4h43	2.30	2
4hu4	2.40	2
4lla	2.50	3
4hl0	2.00	2
4kw3	2.70	2
4ey0	2.80	4
4i6p	2.90	2
4hfy	3.00	2
2etc	3.10	2
2vr9	3.20	3
2etn	3.30	3
3p0l	3.40	4
2xi1	3.50	2
3s0x	3.60	2
2y22	3.70	6
2bbz	3.80	4
1z9x	3.93	3
3bbw	4.00	2
1xdv	4.10	2

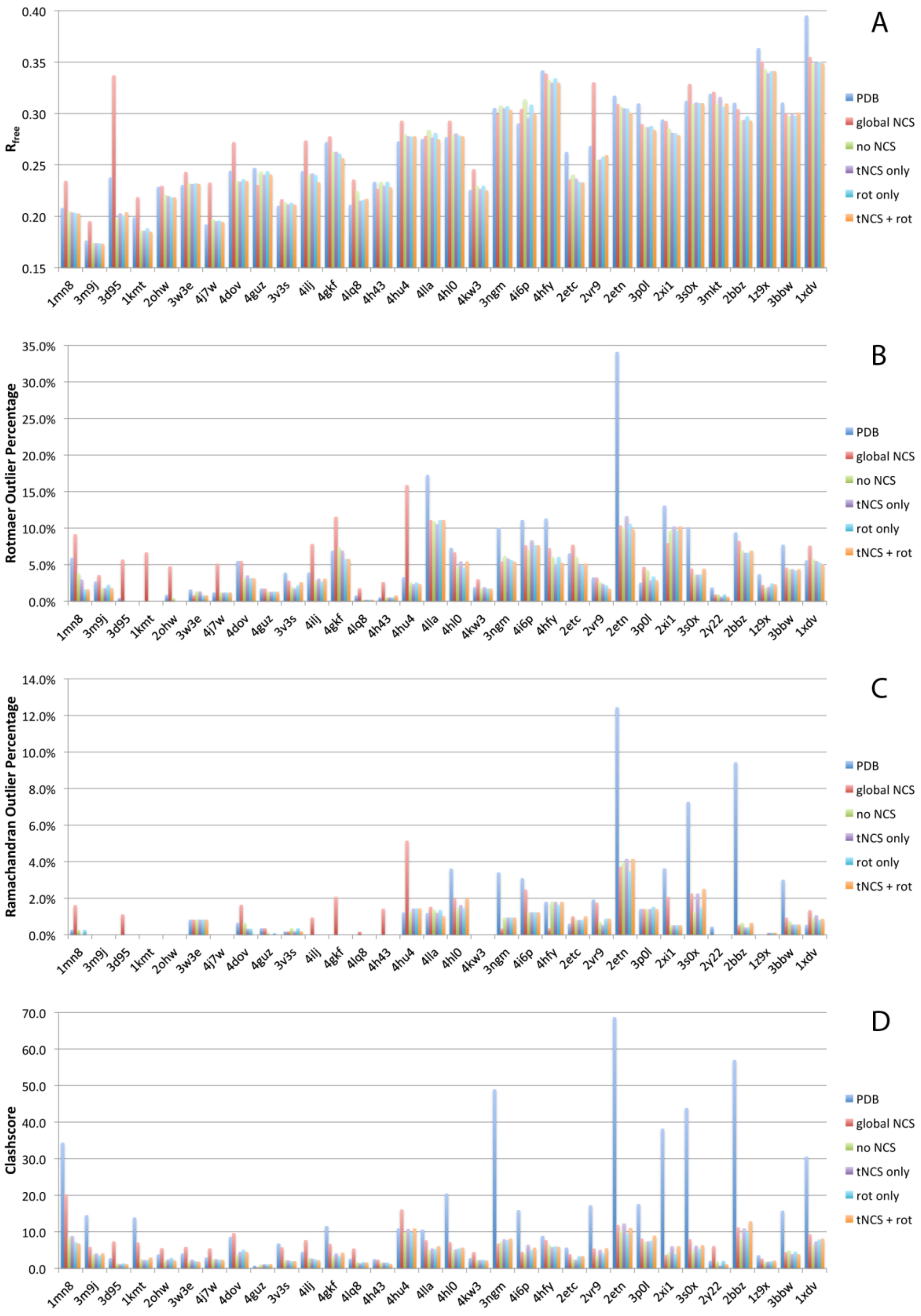
A
R_{free}
0.40
0.35
0.30
0.25
0.20
0.15
1mn8 3m9j 3d95 1kmt 2ohw 3w3e 4j7w 4dov 4guz 3v3s 4ilj 4gkf 4lq8 4h43 4hu4 4lla 4hl0 4kw3 3ngm 4i6p 4hfy 2etc 2vr9 2etn 3p0l 2xi1 3s0x 3mkt 2bbz 1z9x 3bbw 1xdv
PDB
global NCS
no NCS
tNCS only
rot only
tNCS + rot
B
Rotmaer Outlier Percentage
35.0%
30.0%
25.0%
20.0%
15.0%
10.0%
5.0%
0.0%
1mn8 3m9j 3d95 1kmt 2ohw 3w3e 4j7w 4dov 4guz 3v3s 4ilj 4gkf 4lq8 4h43 4hu4 4lla 4hl0 4kw3 3ngm 4i6p 4hfy 2etc 2vr9 2etn 3p0l 2xi1 3s0x 2y22 2bbz 1z9x 3bbw 1xdv
PDB
global NCS
no NCS
tNCS only
rot only
tNCS + rot
C
Ramachandran Outlier Percentage
14.0%
12.0%
10.0%
8.0%
6.0%
4.0%
2.0%
0.0%
1mn8 3m9j 3d95 1kmt 2ohw 3w3e 4j7w 4dov 4guz 3v3s 4ilj 4gkf 4lq8 4h43 4hu4 4lla 4hl0 4kw3 3ngm 4i6p 4hfy 2etc 2vr9 2etn 3p0l 2xi1 3s0x 2y22 2bbz 1z9x 3bbw 1xdv
PDB
global NCS
no NCS
tNCS only
rot only
tNCS + rot
D
Clashscore
70.0
60.0
50.0
40.0
30.0
20.0
10.0
0.0
1mn8 3m9j 3d95 1kmt 2ohw 3w3e 4j7w 4dov 4guz 3v3s 4ilj 4gkf 4lq8 4h43 4hu4 4lla 4hl0 4kw3 3ngm 4i6p 4hfy 2etc 2vr9 2etn 3p0l 2xi1 3s0x 2y22 2bbz 1z9x 3bbw 1xdv
PDB
global NCS
no NCS
tNCS only
rot only
tNCS + rot

Figure S1 Summary of results of re-refinement tests for deposited PDB structures ranging from 1.0-4.1Å resolution. For each metric, the value for the deposited PDB model is given in blue, followed by refinement using global NCS (red), no NCS (green), torsion NCS only (purple), NCS-related rotamer correction (light blue), and torsion NCS plus NCS-related rotamer correction (orange). Results are presented from left to right, highest (1.0Å) to lowest (4.1Å) resolution. A) R_{free} values. B) Rotamer outlier percentage. C) Ramachandran outlier percentage. D) Clashscore.
